# T133. MENTAL HEALTH OF ASYLUM SEEKERS AT THE HUMANITARIAN CAMP IN PARIS

**DOI:** 10.1093/schbul/sby016.409

**Published:** 2018-04-01

**Authors:** Andrea Tortelli, Florence Perquier, Norbert Skurnik, René Wulfman, Marina Ibad-Ramos, Andrei Szoke, Alain Mercuel

**Affiliations:** 1 INSERM; 2 GHT Paris; 3 EPS Maison Blanche

## Abstract

**Background:**

Asylum seeker status is associated to higher prevalence of psychiatric disorders due to not only pre-migration events but also to the trajectory of migration and post- migration adversities. Mental problems and need of care may vary according to these different stages. In France, after the cleaning of the “Calais jungle” in October 2016, most asylum seekers moved to Paris, where they live as homeless. An asylum seeker center (CPA- Centre Premier accueil) was then created a few weeks later, to allow rapid access to asylum seeker procedure, shelter, and medical care (including mental health).

**Methods:**

We will analyze 1- year data (socio-demographic characteristics, migration history, psychiatric symptoms, hospital admissions and medical prescriptions) of about 1000 recently arrived asylum seekers who consulted for psychiatric examination in the refugee camp (CPA). Prevalence and bivariate analyses will be done. Factors associated with mental health problems will be identified using multivariate analyses.

**Results:**

Findings will be discussed in the light of the French/European immigration policy and the organization of the public mental health system.

**Discussion:**

The results of our study will contribute to the identification of mental health problems and correlates at the arrival of asylum seekers in France, in the aim to develop adequate service planning, prevention and support.

